# Protocol for a collaborative meta-analysis of 5-HTTLPR, stress, and depression

**DOI:** 10.1186/1471-244X-13-304

**Published:** 2013-11-12

**Authors:** Robert C Culverhouse, Lucy Bowes, Naomi Breslau, John I Nurnberger Jr, Margit Burmeister, David M Fergusson, Marcus Munafò, Nancy L Saccone, Laura J Bierut

**Affiliations:** 1 Department of Medicine, Washington University School of Medicine, St. Louis, MO, USA; 2 Division of Biostatistics, Washington University School of Medicine, St. Louis, MO, USA; 3 Department of Social Policy and Intervention, University of Oxford, Oxford, UK; 4 Department of Epidemiology and Biostatistics, Michigan State University, East Lansing, MI, USA; 5 Institute of Psychiatric Research, Departments of Psychiatry and Medical and Molecular Genetics, Indiana University School of Medicine, Indianapolis, IN, USA; 6 Molecular and Behavioral Neuroscience Institute, University of Michigan, Ann Arbor, MI, USA; 7 Department of Human Genetics, University of Michigan, Ann Arbor MI, USA; 8 Department of Psychiatry, University of Michigan, Ann Arbor MI, USA; 9 Christchurch Health and Development Study, Department of Psychological Medicine, University of Otago, Christchurch, New Zealand; 10 MRC Integrative Epidemiology Unit, UK Centre for Tobacco Control Studies and School of Experimental Psychology, University of Bristol, Bristol, UK; 11 Department of Genetics, Washington University School of Medicine, St. Louis, MO, USA; 12 Department of Psychiatry, Washington University School of Medicine, Saint Louis, MO, USA

## Abstract

**Background:**

Debate is ongoing about what role, if any, variation in the serotonin transporter linked polymorphic region (5-HTTLPR) plays in depression. Some studies report an interaction between 5-HTTLPR variation and stressful life events affecting the risk for depression, others report a main effect of 5-HTTLPR variation on depression, while others find no evidence for either a main or interaction effect. Meta-analyses of multiple studies have also reached differing conclusions.

**Methods/Design:**

To improve understanding of the combined roles of 5-HTTLPR variation and stress in the development of depression, we are conducting a meta-analysis of multiple independent datasets. This coordinated approach utilizes new analyses performed with centrally-developed, standardized scripts. This publication documents the protocol for this collaborative, consortium-based meta-analysis of 5-HTTLPR variation, stress, and depression.

*Study eligibility criteria:* Our goal is to invite all datasets, published or unpublished, with 5-HTTLPR genotype and assessments of stress and depression for at least 300 subjects. This inclusive approach is to minimize potential impact from publication bias.

*Data sources:* This project currently includes investigators from 35 independent groups, providing data on at least N = 33,761 participants.

The analytic plan was determined prior to starting data analysis. Analyses of individual study datasets will be performed by the investigators who collected the data using centrally-developed standardized analysis scripts to ensure a consistent analytical approach across sites. The consortium as a group will review and interpret the meta-analysis results.

**Discussion:**

Variation in 5-HTTLPR is hypothesized to moderate the response to stress on depression. To test specific hypotheses about the role of 5-HTTLPR variation on depression, we will perform coordinated meta-analyses of *de novo* results obtained from all available data, using variables and analyses determined *a priori*. Primary analyses, based on the original 2003 report by Caspi and colleagues of a GxE interaction will be supplemented by secondary analyses to help interpret and clarify issues ranging from the mechanism of effect to heterogeneity among the contributing studies. Publication of this protocol serves to protect this project from biased reporting and to improve the ability of readers to interpret the results of this specific meta-analysis upon its completion.

## Introduction

Large-scale, collaborative meta-analysis has the potential to clarify complex scientific questions by increasing sample size, harmonizing variables, and conducting uniform analyses. In this paper, we describe the protocol for a collaborative, consortium-based meta-analysis to increase understanding of the role of 5-HTTLPR variation and stress in depression. The publication of this protocol serves several important purposes. First, we document our study protocols, design, and primary analysis decisions prior to conducting and publishing our study of 5-HTTLPR variation, stress, and depression, which will avoid biased reporting later. Second, publication of these details will improve the ability of other researchers to interpret the results of this specific meta-analysis upon its completion. Finally, publishing our detailed protocol may be helpful to future consortia by detailing key analysis strategies and as a standard for transparency and commitment to analytic plans set prior to meta-analysis.

## Background

Both genetic and environmental factors influence depression [1]. As with other psychiatric illnesses, research on the etiology of depression has identified moderate heritability estimates of 40-50% [1-6], yet recent genome-wide association studies (GWAS) have so far been unable to identify robust and replicable loci associated with depression [7]. It has been argued that some of this ‘missing heritability’ is a result of particular genes exerting an influence on risk for depression only under specific environmental conditions, or ‘gene-environment interactions (GxE) [8,9]. One of the most high profile reports of GxE involves a common functional polymorphism (5-HTTLPR) in the promoter region of the serotonin transporter gene (*5-HTT*). This gene encodes an integral membrane protein that transports the neurotransmitter serotonin from synaptic spaces into presynaptic neurons, which terminates the action of serotonin. The repeat length polymorphism has been shown to affect the rate of serotonin uptake [10]. Specifically, the short (S) allele of the 5-HTTLPR is associated with less transcriptional efficiency of the promoter compared to the long (L) allele [11]. Additionally, a single nucleotide substitution (rs25531, A **>** G) within both alleles reduces transcription so that the L_G_ allele becomes functionally equivalent to the S allele [12,13]. Studies have suggested grouping L_G_ with the S allele to increase efficiency in predicting variation in serotonin transporter expression [14], although not all agree on this point.

In 2003, Caspi and colleagues reported evidence of a G×E interaction between 5-HTTLPR variation and stressful life events on depression [15], with nearly 5000 citations to date. Carriers of either one or two copies of the S allele of the 5-HTTLPR were reported to be more likely to develop major depressive disorder, increased depressive symptoms, and suicidality in response to stressful life events and, separately, child maltreatment than individuals homozygous for the L allele. Furthermore, there was evidence of a dose–response relationship, with risk of depression highest amongst those with two copies of the S allele compared to individuals with only one copy in the presence of stress.

In the original report, no main effect of genotype was found. If the genotype exerts an effect only on those exposed to the stressor, i.e. a diathesis-stress model, the lack of main effect may be due to insufficient power [16]. Alternatively, the genotypic effect may be one of differential environmental susceptibility [17], in which the L-carriers are indifferent to the environment, whereas the S allele confers environmental susceptibility, allowing S carriers to benefit more from positive experiences as well as being more sensitive to stress, resulting in no net genotype-depression association irrespective of sample size [18]. Studying a large sample may distinguish these possibilities.

Since the original report of a GxE interaction, hundreds of studies have investigated the combined impact of 5-HTTLPR variation and stress on risk for depression, some of which reported replicating the original findings, while some did not. Meta-analyses, also, have come to quite different conclusions [16,19-22] and various reasons for these differences have been proposed [21,23-25].

Below we discuss 4 key factors that complicate the interpretation of existing results related to the interplay between 5-HTTLPR variation, stress and depression.

1. ***Study design***. One factor complicating interpretation is differences in study design. Sample sizes vary, with the majority modest or small. Underpowered studies, combined with potential publication bias, can lead to an increased risk of Type 1 errors [26]. Sampling strategies vary from population-based methods [15,27] to convenience sampling [28,29]. Different ancestral populations have been included, with a preponderance of samples of European ancestry. The age range of subjects varies, and it has been suggested that GxE effects are most consistently replicated in young adult samples [18].

The studies contributing to our meta-analysis represent a range of study designs, so we will test whether differing study design contributes to the heterogeneity of results.

2. ***Timing of measurements***. Brown and Harris [19] noted that studies that have failed to replicate the GxE between 5-HTTLPR variation and life events have measured the occurrence of stressful life events in the months immediately preceding the depressive outcomes [19]. In contrast, most of the positive replications have been in keeping with the original study by Caspi and colleagues in measuring life events in the five years prior to the outcome. Retrospective recall of adversity over long periods of time may increase the risk of forgetting or discounting of events [30] or lead to a bias due to selective recall when those who are depressed self report [27,31]. When only a lifetime diagnosis of depression is available, information about relative timing of stressors and depression is lost.

Longitudinal studies are largely able to avoid this bias, so our test of longitudinal vs. cross-sectional designs will also, in part, address the issue of timing of stress and depression.

3. ***Type of environmental stressor***. Different stressors have been examined for interaction with 5-HTTLPR variation*.* The most common are broad measures of stressful life events [32,33] and exposure to child maltreatment [34,35]. The method of measurement (self report vs. interviewer), the type of stressors (e.g. chronic vs. acute) and the scale (binary exposed vs. not exposed, frequency as in the original study, or continuous variable) also vary. It has been suggested that the varied methodology in assessing stressful life events in GxE studies may in part explain the inconsistency of findings [18], and in a meta-analysis that differentiated between stressors (child maltreatment, specific stressors, and stressful life events), significant differences between types of stressors were found.

We will therefore perform heterogeneity analyses to account for different types of stressors and measurements of stressors. One such test will be to compare results from the studies that focused on specific stressors (e.g. pregnancy, heart attack, medical internship) to those from studies based on summary measures of diverse stressors (e.g. the LTE-Q).

4. ***Type of outcome***. Some studies used a categorical measure of depression diagnosis (e.g. DSM-IV or the ICD-10) as an outcome, others used a symptom count as continuous outcome, and some used both. Such differences in outcome measures (e.g., continuous versus categorical) result in different assumptions and analytical approaches being used (see for a Discussion [36]).

The role of 5-HTTLPR variation and stress in the development of depression remains a topic of active debate, in part because of the challenges outlined above. Therefore, we are undertaking this collaborative meta-analysis using a standardized protocol to improve understanding of this important issue.

## Study objectives

The primary objective of this study is to increase understanding of the role of 5-HTTLPR variation and stress in depression, where 5-HTTLPR variation is hypothesized as a moderator of the response to stress. To address the complexities of this topic, we will perform a coordinated meta-analysis of all data available from collaborators, using consistent *de novo* analyses and variables determined *a priori*.

In keeping with the original finding by Caspi and colleagues [15], we will test the following main hypothesis.

The risk of depression, evaluated either as a dichotomous diagnosis or as a continuous phenotype, is greater in carriers of the S allele versus those homozygous for the L allele in the presence of exposure to stress.

This main hypothesis will be examined in multiple settings to determine a range of conditions under which the effect might be found. Additional hypotheses (e.g. whether there is a main effect of 5-HTTLPR variation on depression) will be examined to improve our understanding of this complex topic.

## Methods/Design

### Overview

Plans for analysis, definitions of stressors and outcomes, as laid out in this protocol, were determined *a priori* by our study leadership team in consultation with contributing study investigators, who provided input via conference calls and email. Individual study analyses will be performed by the investigators who collected the data, using standardized analysis scripts developed by the coordinating team at Washington University to implement a consistent analytical approach across sites. Results from these analyses will be sent to the coordinating team for meta-analysis. The consortium as a group will review and interpret the results. We have previously developed and successfully implemented this collaborative meta-analysis approach to investigate the genetics of addiction [37-39].

This coordinated meta-analysis approach has several advantages over traditional literature-based meta-analyses arising from the fact that a collaborative meta-analysis is based on *de novo*, consistent analyses of harmonized variables in the primary data. First, the consistent analytic approach and harmonized variables greatly ameliorate the effects of two of the chief causes of heterogeneity between individual studies. In particular, meta-analyzing results derived from differing analytic approaches, such as the use of different covariates, different phenotype definitions, and different analytical models, has a clear potential to bias literature-based meta-analyses [40]. Second, the fact that the results are based on *de novo* analyses allows for the inclusion of previously unpublished data, both from researchers who have not published on the topic before and through updated individual data from research groups who have previously published. Third, coordinated *de novo* analyses of primary data allow a collaborative meta-analysis to conduct secondary analyses that can aid in the interpretation of the main results. Finally, the coordinated meta-analysis approach allows the examination of more homogeneous subsets of the primary data than is possible through literature review.

### Identifying studies

Our goal is to invite participation from all studies with datasets that have the 5-HTTLPR variant genotyped, and assessments of stress and depression, on at least 300 subjects. We set a minimum study size anticipating that the individuals available for our *de novo* analyses would typically be only a subset of the study individuals. Setting an arbitrary study minimum was intended to improve the balance between the heterogeneity introduced by a new study and the increased sample size. It also had the potential to ameliorate bias due to a possible publication bias amongst small studies. One drawback to this approach of requiring large sample size is that it may exclude some studies with the most intensive assessments of stress and depression. Invitations were sent to groups that had previously published on the topic. Additional invitations were sent to groups, published or unpublished on this topic, suggested as a potentially qualifying study by a participating group. The goal of this inclusive approach is to minimize the potential impact of publication bias. The participating collaborative studies are listed in Table 1. All participating studies have approval from their local boards governing ethical conduct of research in human subjects to conduct the agreed upon statistical analyses. A list of the review boards can be found in the acknowledgements. Additional groups that have been invited, but for a variety of reasons are not currently participating, are identified by publications listed in Additional file 1: Table S1.

**Table 1:** Groups participating in the 5-HTTLPR coordinated meta-analysis

**Study**	**Representatives**
ALSPAC	Marcus Munafò, Ricardo Araya, Lucy Bowes
ASPIS	Nicholas Stefanis, Laura Mandelli, Costas N. Stefanis, Alex Hatzimanolis, Alessandro Serretti
ATP	Craig Olsson, Keriann Little
BIGSIBS	Alex Todorov, Vesselin Chorbov
CHDS	David Fergusson, John Horwood
Chinese Academy of Medical Sciences	Xu Qi
CoFaMS	Bernhard T. Baune, Grant Sinnamon, Sarah Cohen-Woods
COGA	John Nurnberger, Jr.
COGEND	Naomi Breslau, Laura Bierut
CTS-LTS and NYSFS	John Hewitt, Brett Haberstick
DeCC	Peter McGuffin, Helen L. Fisher, Sarah Cohen-Woods, Anne Farmer
EPIC-Norfolk	Paul Surtees, Nick Wainwright
ESPRIT	Karen Ritchie, Isabelle Jaussent
G1219	Thalia Eley, Kathryn Lester
Genetic Study of Bipolar Disorder	Frank Bellivier
GENESIS	Philippe Courtet, Alain Malafosse, Emilie Olié
GTP	Kerry Ressler, Bekh Bradley-Davino
Heart and Soul Study	Christian Otte, Mary Whooley
Intern Health Study	Srijan Sen
MARS	Manfred Laucht, Tobias Banaschewski, Daniel Brandeis
MLS	Margit Burmeister, Robert A Zucker, Sandra Villafuerte
MoodInflame (Münster)	Volker Arolt, Bernhard T. Baune
Münster Neuroimaging Study	Udo Dannlowski, Bernhard T. Baune
NESDA	Brenda Penninx, Johannes Smit, Wouter Peyrot
NEWMOOD	Gabriella Juhasz, Bill Deakin, Gyorgy Bagdy, Judit Lazary
NTR (Adult NTR and Young NTR)	Christel Middeldorp, Dorret Boomsma
PATH	Simon Easteal, Kaarin J. Anstey
POUCH	Jeanette Scheid, Claudia Holzman, Nicole Jones
PREDICT-Gene	Blanca Gutiérrez, Jorge Cervilla,
QIMRtwin	Nicholas G. Martin, William L. Coventry, Grant W. Montgomery, Naomi R. Wray
SALVe 2001 and SALVe 2006	Cecilia Åslund, Kent Nilsson
SEBAS	Dana Glei, Noreen Goldman, Maxine Weinstein
SHIP	Hans Jörgen Grabe
TRAILS	Albertine Oldehinkel, Esther Nederhof, Johan Ormel
U. Bologna	Laura Mandelli, Alessandro Serretti
U. Molise	Marco Sarchiapone, Laura Mandelli
VAHCS	George Patton, Craig Olsson, Christina O’Loughlin
Coordinating Team	Robert Culverhouse, Nancy Saccone, Laura Bierut, Amy Horton
Leadership Team	Robert Culverhouse, Laura Bierut, Naomi Breslau, John Nurnberger Jr., Nancy Saccone
External Beta Testers	Lucy Bowes, Sarah Cohen-Woods, Helen Fisher, Brett Haberstick, Marcus Munafò

Inclusion criteria:

●5-HTTLPR variant genotyped.

●Measurement of exposure to stress (including stressful life events and/or maltreatment).

●Measurement of depression (including symptom counts and/or diagnosis).

●Study sample size of 300 or more.

Exclusion criteria:

●Studies where the 5-HTTLPR variant was not directly genotyped.

●Studies with a sample size of less than 300 participants.

### Data management, analysis, and meta-analysis

Multiple existing, independent datasets will contribute *de novo* analysis results for meta-analysis, following coordinated meta-analysis protocols we have developed and implemented previously [37-39]. Extensive meetings between the leaders of the contributing studies have focused on effective ways to harmonize the available variables across the studies (see “Data Harmonization” below) to minimize heterogeneity across the resulting analyses. Following the common protocol, each contributing group will format their data for analysis by a common analysis script. Analyses will be performed by each individual data contributing site, using standardized scripts developed by the coordinating site at Washington University in consultation with the entire consortium. Beta-testing of these scripts will be performed both by the coordinating team and individual study teams. These scripts will ensure consistent analyses across sites. Results will be returned to the coordinating site for meta-analysis. The results of the meta-analyses will be interpreted by the consortium and a collaborative publication reporting the results will be produced.

#### Quality control

The data contributing sites will be responsible for checking the quality of their formatted data (e.g., making sure that values for all variables are credible, confirming the sample size, and checking the number of missing observations for each variable) prior to analysis and in the results returned to the coordinating site. The coordinating site will review the results and work out any anomalies with the data providers, and perform meta-analyses.

#### Data harmonization

Differences in assessment of stress and depression and information regarding the timing of stress and depression, is a major challenge for effectively combining the information from the participating studies. The process of data harmonization began with surveys of the contributing studies to determine what key variables were available in each dataset. Based on this information, the consortium judiciously balanced the effort of trying to include as many studies as possible against the most appropriate variables for analysis. Below we describe the two key variables and approaches selected for study.

1. **Depression:** Many different assessments and evaluations are available for study. Differences in measurement of depression may influence the association results, and as a result, a thorough review of the best measurement to be used in this study was undertaken.

The primary phenotype selected for analysis is a major depressive disorder defined by DSM-IV criteria. This phenotype is one used in the original [15] paper. Additional analyses will be completed using DSM-IV major depressive disorder symptom count, again as in keeping with the original study.

Across the majority of the current contributing datasets, a lifetime diagnosis of DSM-IV major depressive disorder was assessed. For studies where DSM-IV definition of major depressive disorder is not available, a proxy diagnosis will be used. We will track the assessment used and if a proxy diagnostic variable is used, we will examine potential biases introduced by assessment heterogeneity.

2. **Stress:** The studies vary widely on the types of assessments of stress and on the relative timing of the stressful event and the onset of depression. This study will examine two main stress assessments: a narrowly focused stressor, childhood maltreatment (most frequently used assessment among our participating groups: the Childhood Trauma Questionnaire (CTQ) [41]), and a broader defined stressor class, lifetime stressful events (most frequently used assessment: the List of Threatening Events (LTE) [42]). A second dimension we will investigate is timing of stress exposure relative to the assessment of depression. Some analyses will require that timing is known, while others will be broadened to include datasets where the timing is uncertain. A comprehensive list of the stress variables used in the analyses can be found in Additional file 2: Table S2.

We will test our hypotheses in two models, the first using data from a well-characterized, narrowly defined set of subjects selected to most closely resemble those used in the original study by [15] (young adults with no prior history of depression), and a more broadly defined group of subjects.

#### Heterogeneity

Data harmonization requires compromises, and some heterogeneity and biases will surely remain. We will address this issue and improve the interpretation of our primary analyses by testing for heterogeneity across a variety of factors and examining results within and across more homogeneous subsets.

*A priori* group comparisons for heterogeneity will include the following:

●Longitudinal versus cross-sectional studies.

●Different measures of depression (DSM-IV criteria and other measures).

●Different stress exposure scales.

●Interview versus questionnaire reports.

●Self-report versus report by others.

Should heterogeneity be detected, we will report meta-analysis results from homogeneous subgroups as well as results based on all available data. Assessment of heterogeneity across groups of studies and meta-analyses of sub-groups of studies will be carried out by the coordinating team.

### Analysis models

In our exploration of the relationship between 5-HTTLPR variation, stress, and depression, we will try to determine (1) settings in which an effect can be identified and (2) the extent to which an effect can be generalized.

In the original report by Caspi and colleagues [15], a depression outcome at age 26 was assessed using the Diagnostic Interview Schedule [43], yielding a diagnosis of a major depressive episode according to DSM-IV criteria, as well as a continuous measure of depressive symptoms. Stressful life events occurring over a five-year period were assessed retrospectively when study members were aged 26. Subjects with a diagnosis of depression prior to the five-year window were excluded. Stressful events were summed to create a five-level ordinal variable (no life events, one, two, three, and four or more events). Exposure to childhood maltreatment was also examined at three levels (no, probable, and likely maltreatment).

Analyses involved both a logistic regression (multiplicative) model where depression diagnosis was the outcome, and an ordinal least squares regression (additive) model of depressive symptoms. No main effect of genotype was found.

As stated above, our analyses will fall into two main groups of data: a well-characterized, narrowly defined set of subjects similar to those included in the original study, and a broadly defined group of subjects. Each of these groups will be investigated based on two stressor classes: narrow (childhood maltreatment – including physical and sexual abuse as well as physical neglect occurring before age 17) and broad (any lifetime stressful events (e.g. death of family member, job loss, assault, life threatening illness)). The first group of subjects will consist of adults aged 21 to 30, who did not have depression prior to a 5-year window before assessment, and for whom stress exposure is known to have occurred before any current depression. Other than childhood maltreatment, lifetime stressful events will only be considered if they occurred no more than 5 years prior to assessment. The second, broader group of subjects will have no age restriction. Examination of the broader group of subjects will include additional analyses which drop requirements related to the timing of the stressors.

Using these approaches, we intend to balance the competing factors affecting power: sample size and purity of assessments. Analyses of the first class of subjects are designed to most closely match the original report of [15]. By examining the more severe stressor, childhood maltreatment, in a larger sample with no age restriction, our goal is to potentially increase our ability to see the influence of 5-HTTLPR variation if the effect is not limited young adulthood. Our broadest analysis, with stress broadly defined in the largest population, can be expected to maximize power if there is a general effect of 5-HTTLPR variation moderating the impact of stress exposure on depression. If we do not see the influence of 5-HTTLPR variation in this final set of analyses, but it was present in at least one of the previous analyses, we will be able to examine which factors reduce the effect of the association. An important benefit of this design is that we will also examine the degree to which results observed with narrower analyses generalize to the broader analyses, which provides the opportunity to clarify the importance of specific phenotype, stressor, and age requirements.

Under this protocol, data from a particular study may be appropriate for inclusion in one or more of these analysis categories.

### Plan details

The primary model of interest, a focused analysis of 5-HTTLPR variation, consists of two arms (arms A and B) and most closely follows the original report. The analysis scripts will also include pre-specified secondary analyses aimed to aid additional understanding of the primary analysis results and to explore secondary hypotheses.

### Primary analysis 1: analysis of the impact of 5-HTTLPR variation in young adults

In keeping with the original study and following reports that effects of any GxE may be expected to be greatest among young adults [18], we will first restrict our analyses to individuals with outcome measures at age 21–30 years.

Two arms differentiated by stressor will be the co-primary foci for this set of analyses.

#### Arm A: childhood maltreatment

●Narrower Stressor

●No time limit on gap between maltreatment and depression

●Stress exposure assumed to occur before depression, but need not be documented

Childhood maltreatment has been reported as a particular stressor of interest for understanding the relationship between 5-HTTLPR variation and depression. The effects of childhood maltreatment were deemed by the consortium members to have long-lasting adverse effects. As a consequence, no time limit between exposure to childhood maltreatment and adult depression outcome will be required. This is also in keeping with the original study. Furthermore, these analyses will assume that childhood maltreatment antedates the development of depression, so specific timing of when the maltreatment occurred relative to any depressive episodes will not be required.

#### Arm B: stressful life events (including childhood maltreatment)

●Broader Stressor

●Stressors other than childhood maltreatment must occur < 5 years prior to assessment

The second arm will take a broader measure of stress, incorporating both stressful life events and maltreatment history. Because stressful life events other than childhood maltreatment were deemed as less likely to have a lifelong impact, for this analysis, only life stressors occurring within 5 years of the outcome measure will be used. Furthermore, in order to elucidate the issue of causation in the event that an association is observed, only data in which the relative timing of exposure to stressful life events and depression outcome is known will be used. Individuals with a history of depression prior to stress exposure will be excluded from these analyses.

### Key covariates

Several covariates may alter the relationship between 5-HTTLPR variation, stress, and depression. Effects due to sex, age, birth cohort, and genetic ancestry will be investigated in our analysis.

### Sex

Analyses stratified by sex and using sex as a covariate will be conducted.

### Age

Age at interview will be included as a covariate in the primary analyses.

### Birth cohort

Cohort effects will be examined using decade of birth as a covariate. The primary models will not include birth cohort as a covariate.

### Genetic ancestry

Our analyses will stratified by genetic ancestry (European, Asian, African, Pacific Island, and Admixed European-African). The majority of data is from participants of European ancestry and there may be limited power to detect ethnic heterogeneity in the results. If there is sufficient power to effectively test for heterogeneity and none is found, then a meta-analysis across all ancestry groups will be evaluated.

### Statistical models for primary analysis 1

#### Arm A analysis

##### Analysis model 1A1

Moderated logistic regression (dichotomous phenotypes).

$$D=\mu +\beta_{1}M+\beta_{2}G+\beta_{3}\left(M\times G\right)+\sum_{i=4}^{n}\beta_{i}\mathrm{co}v_{i}$$

where

D = diagnosis of DSM-IV major depressive disorder.

M = dichotomous measure of exposure to childhood maltreatment.

G = 5-HTTLPR genotype.

In this analysis, model fit using a likelihood ratio test is compared with and without the interaction term to evaluate whether there is statistically significant evidence for the interaction. Our primary analysis will use an additive coding for genotype based on the number of S alleles carried and lifetime diagnosis of depression.

Secondary analyses will evaluate whether alternate codings for genotype (such as adding a dominance factor) provide a significantly better fit to the data and the possibility of a main effect of 5-HTTLPR genotype on depression, and investigate both current depression and any depression as alternate outcomes.

##### Analysis model 1A2

Moderated linear regression (quantitative phenotypes).

$$D_{\mathrm{quant}}=\mu +\beta_{1}M_{\mathrm{quant}}+\beta_{2}G+\beta_{3}\left(M_{\mathrm{quant}}\times G\right)+\sum_{i=4}^{n}\beta_{i}{\mathrm{cov}}_{i}$$

where

D_*quant*_ = DSM-IV symptom count major depressive disorder.

M_*quant*_ = quantitative measure of exposure to childhood maltreatment.

G = 5-HTTLPR genotype.

This analysis will parallel the description of Model 1A1.

#### Arm B analysis

Analysis Models 1B1 and 1B2 will be analogous to 1A1 and 1A2, but substituting stressful life events, S, as the stressor. Stressors other than childhood maltreatment must have occurred no more than 5 years prior to the assessment of depression to be included.

These two arms of Primary Analysis 1 will most closely replicate the analyses in the original report. If, as has been suggested, the GxE effect is strongest for childhood maltreatment [19] or is seen primarily in young adults following recent stress exposure [18] this set of analyses will maximize statistical power.

### Primary analysis 2: analysis of the impact of 5-HTTLPR variation across all ages

Our second set of primary analyses will involve larger sample sizes, including children and adults of all ages. The increase in sample size will result in increased power if there is a broad genetic association between 5-HTTLPR genotypes, stress, and depression. However, this comes at a cost; in these analyses, we give up the opportunity to investigate whether stress preceding depression was a potential cause of the depression, as relative timing of stress and depression may not be known, and thus will not be included in the models.

Parallel to Primary Analysis 1, these analyses will also utilize two co-primary arms differentiated by stressor.

#### Arm A: childhood maltreatment

●Narrower Stressor.

●No time limit on gap between maltreatment and depression.

●Stress exposure assumed to occur before depression, but need not be documented.

As noted above, childhood maltreatment has been reported as a particular stressor of interest for understanding the relationship between 5-HTTLPR variation and depression. This portion of the analysis differs from the first primary analysis Arm A in that there is no limit on the ages of the subjects at the time of interview.

#### Arm B: stressful life events (including childhood maltreatment)

●Broader Stressor

●Stressors occurring < 5 years prior to assessment when information available.

●Stressor timing unknown will not be reason for exclusion.

This arm of the analyses will include a series of nested analyses. The first will be similar to Arm B of the first primary analyses, except that there is no restriction on the ages of the subjects. Additional analyses will further reduce the restrictions on the included data (e.g. timing need not be known) as we attempt to determine what are the broadest general conditions under which an effect can be detected. As we loosen the restrictions, the sample size will increase allowing the possibility of detecting weaker associations in a broader setting.

### Statistical models for primary analysis 2

These analyses will consist of 3 sets of analyses nested based on the subjects that are included.

### Analysis models 2A1a through 2B2a

These are identical to Models 1A1 through 1B2 except that subjects are not restricted to be between age 21 and 30.

### Analysis models 2B1b and 2B2b

These are identical to Models 2A1a through 2B2a except that the timing of the stressful life events relative to depression may be unknown. (Because childhood maltreatment is assumed to occur prior to the first major depressive episode, there are no models 2A1b and 2A2b.)

### Analysis model 2C

Our broadest analysis will estimate allele frequencies among the cells of a 2×2 table defined by stress exposure and lifetime diagnosis of major depression, followed by a determination of whether there is evidence of a direct or interaction association between 5-HTTLPR variation and depression.

### Secondary models

As noted above, in addition to the moderated regression models described in the 11 primary analysis models (1A1 through 2C), we will examine a variety of secondary models to help us interpret the results of our primary results. Included in these will be analyses where only stresses other than childhood maltreatment are considered. Secondary analyses will also include both alternate statistical models and analyses of more narrowly defined subsets of subjects (e.g. subjects from longitudinal studies, subjects for which the SNP occurring on the L allele has been genotyped).

Among these secondary analyses will be traditional logistic and linear regression models parallel to the moderated regression models 1A1 through 2B2b but without the interaction terms, as illustrated below.

#### Secondary Models S1A1 & S1B1

Traditional logistic regression (dichotomous variables).

$$\left(\text{S1A1}\right)\;D=\mu +\beta_{1}M+\beta_{2}G+\sum_{i=3}^{n}\beta_{i}\mathrm{co}v_{i}$$

$$\text{and}\;\left(\text{S1B1}\right)\;D=\mu +\beta_{1}S+\beta_{2}G+\sum_{i=3}^{n}\beta_{i}\mathrm{co}v_{i}$$

Results from previous studies (e.g. [16]) suggest that there is likely no main effect of 5-HTTLPR variation on depression and as a result, this is not part of our primary analysis. Nonetheless, our secondary analyses will evaluate the possibility of a main effect, both in isolation and as part of larger interaction models (cf. models S1A1 and S1B1 above) for completeness.

### Variables included in the analysis datasets

Additional file 2: Table S2 is a comprehensive list of the variables used in the analyses. The studies will generally contain only a subset of the complete list and therefore each dataset will participate in only a subset of the proposed analyses. In addition to the variables contained in the datasets, we will gather additional information about the datasets to allow us to evaluate particular sources of heterogeneity through meta-analyses of refined subsets of the datasets.

Variables gathered about each dataset:

1. Genetic ancestry (datasets are stratified by ancestry).

2. Cross-sectional or longitudinal.

3. Information gathered via interview or questionnaire.

4. Information on stress and depression is based on current or lifetime reports.

### Additional secondary analyses

As indicated above, a variety of secondary, interpretive analyses are planned. In addition to the alternate genotype codings and covariates listed above, tests of heterogeneity between datasets are expected to lead to secondary meta-analyses of results from more homogeneous datasets. In particular, it has been argued that many studies that have failed to replicate the original studies lacked in-depth environmental measures [30]. We will investigate potential differences in results between brief, self-report questionnaire measures of stressful life events and face-to-face interviews as well as differences due to the type of study and the diagnostic system used for assessment. Therefore, in addition to subsets identified through tests for heterogeneity, stratified meta-analyses based on the following factors are planned:

1. Genetic Ancestry (e.g. European, Asian, Pacific Island, Admixed European-African).

2. Study Type (e.g. cross-sectional versus longitudinal data collection).

3. Assessment type (e.g. interview versus questionnaire, diagnostic system).

4. Stress and depression phenotypes are based on current state or lifetime reports.

One source of heterogeneity within an individual study is the source of stress. A subset of our participating groups minimized this particular source of heterogeneity by ascertaining subjects that had all been exposed to a uniform stressor (e.g. pregnancy, military conscription, medical residency, coronary heart disease). Because the primary hypothesis supposes that the effect of genotype can be seen most strongly (or only) in subjects exposed to stress, analyses including only these datasets may have particular power to detect the hypothesized effect.

### Statistical software

All analyses will be conducted using R due to its computational flexibility and free availability.

### Group collaboration

The leadership team is responsible for the project’s management decisions and the daily management of this collaboration. A series of conference calls with the contributing investigative teams discussed and reviewed many issues in the design of the meta-analysis. The leadership team, in collaboration with individual study sites, developed the initial protocol based on these discussions. The coordinating team at Washington University developed the analysis scripts and will perform the meta-analysis. External beta testers for the scripts were essential for the production and distribution of high-quality scripts to the consortium members.

### Ethics

All participating research groups have appropriate ethics approval for the use of their data in the proposed secondary analyses. A listing of the participating studies, the names of the study representatives collaborating on this project, and the organizations providing ethical review for the studies can be found in Additional file 3: Table S3. The Human Research Protection Office of Washington University in St. Louis has stated that this collaborative meta-analysis paradigm does not require ethical approval beyond what has been provided to the individual participating groups.

## Discussion

### Limitations

In this study, our emphasis is on harmonizing the analysis to maximize the number of studies to be included. The hope is that a large sample size will overcome limitations due to heterogeneity in measurements of depression and environment. This assumption is supported by many GWAS meta-analyses and studies which find many more genetic associations even when compromising on phenotyping, e.g. by considering anyone with a clozapine prescription a case, rather than a formal schizophrenia diagnosis (e.g. [44,45]). However, there are limitations to this approach. Although the analysis will be uniform, neither the phenotype nor the environment was measured in a consistent fashion in all studies.

Second, the diathesis-stress model would predict that an alternative approach might be to study a population that was uniformly exposed to a specific stressor. A minority of the studies in our consortium are of this type (e.g. ASPIS (military conscription), Heart and Soul (coronary heart disease), Intern Health Study (medical internship), POUCH (pregnancy)).

Third, limiting the sample size to 300 was done for practical reasons. On the other hand, the small published studies were in their majority those with positive findings [21]). Small samples may be more likely subject to publication bias [23], but also tend to be assessed more carefully, and hence this criterion may exclude some of the best characterized samples.

Fourth, although we will perform a number of specific heterogeneity tests, it is impossible to test all combinations.

Hence, although we will include many possible confounds and expect to have power to detect a robust effect of genotype, a negative finding does not exclude the possibility that a genetic effect exists that is very sensitive to timing or to the method of measuring depression or stress.

## Summary

There is an ongoing debate about what role, if any*,* 5-HTTLPR variation plays in depression. Various studies have argued for (e.g., [15]) or against (e.g., [46]) the proposition that an interaction between 5-HTTLPR variation and stressful life events alters the subsequent risk of depression. Both individual studies and meta-analyses of published results have come to differing conclusions. Numerous issues may contribute to the conflicting results including: heterogeneity among studies (varying study populations, varying definitions of depression, varying measures of stress, prospective versus retrospective assessments, varying analytical models); issues regarding timing of stress and depression; underpowered samples; and publication bias. To address the numerous complexities of the topic of the potential impact of 5-HTTLPR variation on depression, hypothesized as a moderator of the response to stress, we have designed a coordinated meta-analysis that performs consistent, *de novo* analyses of all available primary data, utilizing variables that are harmonized across the datasets and with analyses that were determined *a priori*. These analyses will be implemented in a set of primary analyses, arising from the original report of a GxE interaction involving 5-HTTLPR variation, and supplemented by secondary analyses intended to aid in the interpretation of the main findings and to address numerous issues related to possible heterogeneity between the contributing studies. The risk of data mining and of type 1 errors in large, multidimensional datasets such as ours is great, and there is a clear need for consensus on best practice *before* analyses are conducted. It is our hope that by publishing this protocol in advance of all analyses being conducted, we will minimize potential biases.

## Nomenclature

●5-HTT: Serotonin transporter gene.

●Official name: *SLC6A4*, location: 17q11.2. Also known as HTT; 5HTT; OCD1; SERT; 5-HTT; SERT1; hSERT.

●Summary: This gene encodes an integral membrane protein that transports the neurotransmitter serotonin from synaptic spaces into presynaptic neurons. The encoded protein terminates the action of serotonin and recycles it in a sodium-dependent manner. This protein is a target of psychomotor stimulants, such as amphetamines and cocaine, and is a member of the sodium:neurotransmitter symporter family.

●5-HTTLPR: serotonin-transporter-linked polymorphic region. Summary: A repeat length polymorphism in the promoter of the 5-HTT gene that has been shown to affect the rate of serotonin uptake. The short (“s”) allele is associated with lower transcriptional efficiency of the promoter compared to the long (“l”) allele.

●GxE: Gene-environment interaction.

## Competing interests

The authors have no competing interests to declare.

## Authors’ contributions

LJB and NB initially conceived of the study. RCC recruited the participating studies, led the protocol design discussions, and drafted the protocol. All authors contributed to the design of the protocol. LB and RCC drafted the manuscript. All authors helped edit the manuscript and approved the final manuscript.

## Pre-publication history

The pre-publication history for this paper can be accessed here:

http://www.biomedcentral.com/1471-244X/13/304/prepub

## Supplementary Material

Additional file 1: Table S1 Invited groups that are not currently participating. Click here for file

Additional file 2: Table S2 Variables used for the analyses. Click here for file

Additional file 3: Table S3 Human Research Protection Review for participating studies. Click here for file
