# Supplementary material for: Protocol for a collaborative meta-analysis of 5-HTTLPR, stress, and depression
Source: BMC Psychiatry. 2013 Nov 12;13:304. doi: 10.1186/1471-244X-13-304 (PMC3840571; doi:10.1186/1471-244X-13-304)
Supplement: Additional file 1: Table S1 — Invited groups that are not currently participating. [file 1471-244X-13-304-S1.doc]

**Additional file 1: Table S1.** Invited groups that are not currently participating

| Key Paper | |
| --- | --- |
| Influence of life stress on depression: Moderation by a polymorphism in the 5-HTT gene | Caspi et al. 2003 |
| The interaction of stressful life events and serotonin transporter polymorphism in the prediction of episodes of major depression: a replication. | Kendler et al. 2005 |
| Influence of Serotonin Transporter Gene Polymorphism on Depressive Symptoms and Future Cardiac Events After Acute Myocardial Infarction. | Nakatani et al. 2005 |
| Stress-Related Negative Affectivity and Genetically Altered Serotonin Transporter Function | Jacobs et al. 2006 |
| Interactions of child maltreatment and serotonin transporter and monoamine oxidase A polymorphisms: depressive symptomatology among adolescents from low socioeconomic status backgrounds | Cicchetti et al. 2007 |
| Interactions between life stressors and susceptibility genes (5-HTTLPR and BDNF on depression in Korean elders. | Kim, JM et al. 2007 |
| Early adversity and 5-HTT/BDNF genes: new evidence of gene–environment interactions on depressive symptoms in a general population | Aguilera et al. 2009 |
| Modification by Two Genes of Associations Between General Somatic Health and Incident Depressive Syndrome in Older People | Kim et al. 2009 |
| Genetic predictors of depressive symptoms in cardiac patients | McCaffery et al. 2009 |
| Transition from stress sensitivity to a depressive state: longitudinal twin study | Wichers et al. 2009 |
| No association between polymorphism of serotonin transporter gene and depression in Parkinson's disease in Chinese. | Zhang et al. 2009 |
| Influence of serotonin transporter promoter variation on the effects of separation from parent/partner on depression | Lavebratt et al. 2012 |
| HPA Axis Reactivity: A Mechanism Underlying the Associations Among 5-HTTLPR, Stress, and Depression | Gotlib et al. 2008 |
