# Supplementary material for: Protocol for a collaborative meta-analysis of 5-HTTLPR, stress, and depression
Source: BMC Psychiatry. 2013 Nov 12;13:304. doi: 10.1186/1471-244X-13-304 (PMC3840571; doi:10.1186/1471-244X-13-304)
Supplement: Additional file 2: Table S2 — Variables used for the analyses. [file 1471-244X-13-304-S2.doc]

**Additional file 2: Table S2. Variables used for the analyses**

|  | **Variable** | **Coding** | **Description of variable** |
| --- | --- | --- | --- |
| 1 | Id | alpha numeric variable | Unique value for each subject |
| **Demographic Variables** | | | |
| 2 | Sex | 0=female  1=male  -9 = missing | Sex of subject |
| 3 | Age | Decimal > 0  -9 = missing | Age of subject at interview |
| 4 | Birth_decade | 4 digit decade of birth  e.g. 1950 for birthdates from  Jan 1, 1950 to Dec 31, 1959 | 4 digit decade of birth |
| **Genetic Variables** | | | |
| 5 | Gen5 | LL = 2 copies of the long allele  LS = heterozygote  SS = 2 copies of the short allele  -9 = missing | L=long allele,  S=short allele of *5HTTLPR* |
| 6 | rs25531 | 0 = 0 copies of the G allele  1 = 1 copy of G  2 = 2 copies of G  -9 = missing | # of G alleles for rs25531  **Note:** The G allele for rs25531 occurs most frequently with the L allele for *5HTTLPR*.  If you know the phasing for Gen5 and rs25531, please list the number copies of the LG allele for this variable. If you do not know the phasing, we will assume that ambiguous haplotypes (double heterozygotes) are SA-LG |
| **Depression Variables** | | | |
| 7 | Dep_dx_curr | 0 = not current diagnosis of major depressive disorder  1 = current diagnosis of major depressive disorder  -9 = missing | Current depression diagnosis  Subject has a diagnosis of major depression at time of interview (previous 6 months) |
| 8 | Dep_quant_curr | ≥ 0 if assessed  -9 = missing | Current quantitative depression scale  If you have DSM-IV symptom count at time of interview (previous 6 months), use it for this variable.  Otherwise, use your own scale, but if necessary add a constant to all values so all scored values ≥ 0 |
| 9 | Dep_dx_life | 0 = no lifetime diagnosis (dx) of major depressive disorder  1 = positive lifetime dx of major depressive disorder  -9 = missing | Lifetime depression diagnosis  Subject has at some time during lifetime qualified for a diagnosis of major depressive disorder |
| 10 | Dep_quant_life | ≥ 0 if assessed  -9 = missing | Lifetime quantitative depression scale  If subject has a lifetime diagnosis of major depressive disorder, give symptom count when first met diagnosis of major depressive disorder.  Otherwise, give maximum lifetime value.  If you have DSM-IV symptom count, use it for this variable.  Otherwise, use your own scale, but if necessary add a constant to all values so that all scored values are ≥ 0 |
| 11 | Dep_curr_ao | Decimal integer  -9 = missing | Age of onset of current depression episode  **(If not currently depressed, = age at interview)^1^** |
| 12 | Dep_life_ao | Decimal integer  -9 = missing | Age of onset of first depression episode during lifetime  **(If never depressed, = age at interview) ^1^** |
| **Childhood Maltreatment Variables** | | | |
| 13 | CTQ_EA | 5-25 (CTQ^2^ emotional abuse)  -9 if missing | CTQ sub-score for emotional abuse |
| 14 | CTQ_PA | 5-25 (CTQ physical abuse)  -9 if missing | CTQ sub-score for physical abuse |
| 15 | CTQ_SA | 5-25 (CTQ sexual abuse)  -9 if missing | CTQ sub-score for sexual abuse |
| 16 | CTQ_EN | 5-25 (CTQ emotional neglect)  -9 if missing | CTQ sub-score for emotional neglect |
| 17 | CTQ_PN | 5-25 (CTQ physical neglect)  -9 if missing | CTQ sub-score for physical neglect |
| 18 | Child_mal_exp | 0 = not exposed to childhood maltreatment  1 = exposed to childhood maltreatment  -9 = unknown | Only include this variable if at least one of CTQ subscales for Physical Abuse, Sexual Abuse, and Physical Neglect is not available ^3^  (variables CTQ_PA, CTQ_SA, CTQ_PN) |
| 19 | Child_mal_quant | ≥ 0 if assessed  -9 if missing | Only include this variable if at least one of the CTQ subscales for Physical Abuse, Sexual Abuse, and Physical Neglect is not available ^3^  (variables CTQ_PA, CTQ_SA, CTQ_PN) |
| **Other Stress Variables (Life stress)** | | | |
| 20 | Life_stress_exp | 0 = not exposed  1= exposed  -9 missing | Dichotomous stress exposure  (NOT including childhood maltreatment)  If you are using LTE-Q, please require at least 2 events to be considered exposed |
| 21 | Life_stress_quant | ≥ 0 if assessed  -9 if missing | If you have LTE-Q available, please use that score |
| 22 | Age_last_stress_before_first_dep | Decimal years if known  -9 if missing | Most recent age before first depression when subject was exposed to Life stress (other than childhood maltreatment).  cf var 20, Life_stress_exp  If never depression OR never Life stress exposure, then list current age^1^ |
| 23 | Age_last_stress_before_curr_dep | Decimal years if known  -9 if missing | Most recent age before current depression when subject was exposed to Life stress (other than childhood maltreatment).  If no current depression diagnosis OR never Life stress exposure, then list current age^1^ |
| **Additional timing variables (create ONLY if variables 11, 12, 22, or 23 are missing)** | | | |
| 24^4^ | Tbefore_life | 0 = Depression occurred before first exposure to Life stress  1 = Exposure to Life Stress occurred before first depression,  **OR** no stress exposure,  **OR** no lifetime depression diagnosis  -9 = unknown timing | **Life stress occurred before first depressive episode**  **Only include this variable** if one of variable 9 and 10 are present **AND**  variables 12 or 22 are missing  v9 = lifetime depression diagnosis  v10 = lifetime quant depression  v12 = age of onset of lifetime depression  v22 = age of last stress before first depression |
| 25^3^ | T5yr_life | 0 = All Life stress occurred more than 5 years prior to  a: first depression  **OR**  b: interview if no lifetime diagnosis of depression  1 = Known no Life Stress exposure in preceding 5 years or some stress exposure occurred within 5 years of  a: first depression  **OR**  b: interview if no lifetime diagnosis of depression  -9 = unknown distance, relative to a 5 year window, between Life stress and  a: first depression  **OR**  b: interview if no lifetime diagnosis of depression | **Life stress occurred before, but not more than 5 years prior to first depressive episode**  **Only include this variable** if one of variables 9 and 10 are present.  **AND**  variables 12 or 22 are missing. |
| 26^3^ | T6mo_life | 0 = All Life Stress occurred more than 6 months prior to  a: first depression  **OR**  b: interview if no depression dx  1 = Known no Life Stress exposure in 6 months preceding or some stress exposure occurred within 6 months of  a: first depression  **OR**  b: interview if no lifetime depression diagnosis  -9 = unknown distance, relative to a 6 month window, between Life stress and  a: first depression  **OR**  b: interview, if no depression | **Life stress occurred prior to, but within 6 months of first depressive episode**  **Only include this variable** if one of variables 9 and 10 are present.  **AND**  variables 12 or 22 are missing. |
| 27^3^ | Tbefore_curr | 0 = Current depression occurred before first exposure to Life stress  1 = Known no Life Stress exposure, exposure occurred before current depression, or no current depression diagnosis  -9 = unknown timing | **Life stress occurred before current depressive episode**  **Only include this variable** if one of variables 7 or 8 is present **AND**  variables 11 or 23 are missing.  v7= current depression diagnosis  v8= current quantitative depression  v11=age of onset of current depression  v23=age of last stress before current depression |

| 28^3^ | T5yr_curr | 0 = all Life stress occurred more than 5 years prior to  a: current depression  **OR**  b: interview if no current diagnosis of depression  1 = Known no Life Stress exposure in preceding 5 years or some life stress exposure occurred within 5 years prior to  a: first depression  **OR**  b: interview if no current diagnosis of depression  -9 = unknown if timing falls within 5 years of interview or occurrence of stress unknown | **Life stress occurred before, but not more than 5 years prior to current depressive episode**  **Only include this variable** if one of variables 7 or 8 is present  **AND**  variables 11 or 23 are missing. |
| --- | --- | --- | --- |
| 29^3^ | T6mo_curr | 0 = All Life stress occurred more than 6 months prior to  a: current depression  **OR**  b: interview if no current diagnosis of depression  1 = Known no Life Stress exposure in preceding 6 months or some life stress exposure occurred within 6 months prior to  a: current depression  **OR**  b: interview if no current diagnosis of depression  -9 = unknown if timing falls within 6 months of interview or occurrence of stress unknown | **Life stress occurred prior to, but within 6 months of current depressive episode**  **Only include this variable if**  one of variables 7 or 8 is present  **AND**  variables 11 or 23 are missing. |

^1^CTQ = Childhood Trauma Questionnaire

^2^ This is so that R will not eliminate such subjects from appropriate analyses

^3^ Primary childhood maltreatment variable is based on these three variables

^4^ NOTE: Variables 24-29 ONLY apply to stress OTHER THAN childhood maltreatment
