# Supplementary material for: Protocol for a collaborative meta-analysis of 5-HTTLPR, stress, and depression
Source: BMC Psychiatry. 2013 Nov 12;13:304. doi: 10.1186/1471-244X-13-304 (PMC3840571; doi:10.1186/1471-244X-13-304)
Supplement: Additional file 3: Table S3 — Human Research Protection Review for participating studies. [file 1471-244X-13-304-S3.doc]

**Additional file 3: Table S3. Human Research Protection Review** for participating studies

| **Full Study Name** | **Study** | **Participating representatives** | **Human Research Protection Organization** |
| --- | --- | --- | --- |
| Avon Longitudinal Study of Parents and Children | ALSPAC | Marcus Munafò, Ricardo Araya, Lucy Bowes | ALSPAC Ethics and Law Committee and Local Research Ethics Committees |
| Athens Study of Psychosis Proneness and Incidence of Schizophrenia, University Mental Health Research Institute, Athens, Greece | ASPIS | Nicholas Stefanis, Laura Mandelli, Costas N. Stefanis, Alex Hatzimanolis, Alessandro Serretti | Bioethics and Medical Deontology Committee of the University Mental Health Research Institute (UMHRI) in Athens Greece |
| Australian Temperament Project | ATP | Craig Olsson, Keriann Little | The University of Melbourne Human Research Committee |
| BIGSIBS | BIGSIBS | Alex Todorov, Vesselin Chorbov | Washington University Human Research Protection Office |
| Christchurch Health and Development Study | CHDS | David Fergusson, John Horwood | Southern Health and Disability Ethics Committee |
| Chinese Academy of Medical Sciences | Chinese Academy of Medical Sciences | Xu Qi | Ethics Committee of Xi Yuan Hospital of China Academy of Chinese Medical Sciences |
| Cognitive Function and Mood Study (CoFaMS), University of Adelaide | CoFaMS | Bernhard T. Baune, Grant Sinnamon, Sarah Cohen-Woods | University of Adelaide Ethics Committee |
| Collaborative Study on the Genetics of Alcoholism | COGA | John Nurnberger, Jr. | Washington University Human Research Protection Office |
| Collaborative Genetic Study of Nicotine Dependence | COGEND | Naomi Breslau, Laura Bierut | Washington University Human Research Protection Office |
| Community Twin and Longitudinal Twin Samples National Youth and Family Study | CTS-LTS and NYSFS | John Hewitt, Brett Haberstick | Colorado University-Boulder's Institutional Review Board |
| Depression Case-Control study | DeCC | Peter McGuffin, Helen L. Fisher, Sarah Cohen-Woods, Anne Farmer | Joint South London and Maudsley and the Institute of Psychiatry Research Ethics Committee |
| European Prospective Investigation into Cancer – Norfolk | EPIC-Norfolk | Paul Surtees, Nick Wainwright | Norwich District Ethics Committee |
| Enquête de Santé Psychologique – Risques, Incidence et Traitement Project | ESPRIT | Karen Ritchie, Isabelle Jaussent | Comité de protection des personnes (CPP) Sud Ouest et Outre-mer I |
| The Genesis 12-19 Study | G1219 | Thalia Eley, Kathryn Lester | South London and Maudsley and the Institute of Psychiatry NHS Research Ethics Committee |
| Genetic Study of Bipolar Disorder | Genetic Study of Bipolar Disorder | Frank Bellivier | Comité de Protection des Personnes (CPP) Sud Ouest et Outre-mer I |
| GENESIS : GEnetics, NEuropsychology, functional neuroImaging of Suicidal behaviour | GENESIS | Philippe Courtet, Alain Malafosse, Emilie Olié | Comité de Protection des Personnes "Sud-Méditerranée IV", Montpellier |
| Grady Trauma Project | GTP | Kerry Ressler, Bekh Bradley-Davino | Emory University Institutional Review Board |
| Heart and Soul Study | Heart and Soul Study | Christian Otte, Mary Whooley | Human Research Protection Program Committee on Human Research, University of California, San Francisco |
| Intern Health Study | Intern Health Study | Srijan Sen | IRBMED Univ. of Michigan |
| Mannheim Study of Children at Risk | MARS | Manfred Laucht, Tobias Banaschewski, Daniel Brandeis | Heidelberg University, Ethics Committee of the Medical Faculty |
| Michigan Longitudinal Study | MLS | Margit Burmeister, Robert A Zucker, Sandra Villafuerte | IRBMED Univ. of Michigan |
| MoodInflame (Münster), Universität Münster | MoodInflame (Münster) | Volker Arolt, Bernhard T. Baune | University of Münster Ethics committee |
| Münster Neuroimaging Study, Universität Münster | Münster Neuroimaging Study | Udo Dannlowski, Bernhard T. Baune | University of Münster Ethics committee |
| Netherlands Study on Depression and Anxiety | NESDA | Brenda Penninx, Johannes Smit, Wouter Peyrot | VU University Medical Center |
| New molecules in mood disorders: a genomic, neurobiological and systems approach in animal models and human disorder | NEWMOOD | Gabriella Juhasz, Bill Deakin, Gyorgy Bagdy, Judit Lazry | Budapest: Scientific and Research Ethics Committee of the Medical Research Council (ETT TUKEB)  Manchester: North Manchester Local Research Ethics Committee |
| Netherlands Twin Register (Adult NTR and Young NTR) | ANTR and YNTR | Christel Middeldorp, Dorret Boomsma | VU University Medical Center |
| Personality And Total Health through life | PATH | Simon Easteal, Kaarin J. Anstey | ANU Human Research Ethics Committee |
| Pregnancy Outcomes and Community Health Studies | POUCH | Jeanette Scheid, Claudia Holzman, Nicole Jones | Michigan State University Human Research Protection Program |
| PREDICT-Gene | PREDICT-Gene | Blanca Gutiérrez, Jorge Cervilla, | Comité ética en investigación humana; University of Granada, Spain |
| Queensland Institute of Medical Research Twin and Family Study | QIMRtwin | Nicholas G. Martin, William L. Coventry, Grant W. Montgomery,  Naomi R. Wray | Queensland Institute of Medical Research - Human Research Ethics Committee |
| Centre for Clinical Research, Västerås, Sweden. Survey of Adolescent Life in Vestmanland 2001 and 2006 | SALVe 2001 and SALVe 2006 | Cecilia Åslund, Kent Nilsson | The regional ethical review board of Uppsala University |
| Social Environment and Biomarkers of Aging Study | SEBAS | Dana Glei, Noreen Goldman, Maxine Weinstein | Institutional Review Boards at: Georgetown University in Washington, D.C., Princeton University in Princeton, NJ. Bureau of Health Promotion, Department of Health in Taichung, Taiwan |
| Study of Health in Pomerania | SHIP | Hans Jörgen Grabe | Ethical Board of the University of Greifswald |
| TRacking Adolescents’ Individual Lives Survey | TRAILS | Albertine Oldehinkel, Esther Nederhof, Johan Ormel | Dutch Central Committee on Research involving Human Subjects (CCMO) |
| University of Bologna, Institute of Psychiatry, Department of Biomedical and Neuromotor Sciences, Bologna, Italy | University of Bologna | Laura Mandelli, Alessandro Serretti | Ethical committee of the Scientific Institute San Raffaele hospital, Milan, Italy |
| University of Molise, Department of Health Sciences, Campobasso, Italy | University of Molise | Marco Sarchiapone, Laura Mandelli | Ethics committee of the University of Molise |
| Victoria Adolescent Health Care Study | VAHCS | George Patton, Craig Olsson, Christina O'Loughlin | Royal Children's Hospital Human Research Ethics Committee |
